# Exclusive Breastfeeding Knowledge, Intention to Practice and Predictors among Primiparous Women in Enugu South-East, Nigeria

**DOI:** 10.1155/2019/9832075

**Published:** 2019-01-03

**Authors:** Chikaodili N. Ihudiebube-Splendor, Chinyelu B. Okafor, Agnes N. Anarado, Nonyelum N. Jisieike-Onuigbo, Anthonia U. Chinweuba, Ada C. Nwaneri, Joyce C. Arinze, Paulina C. Chikeme

**Affiliations:** ^1^Department of Nursing Sciences, University of Nigeria Enugu Campus, Nigeria; ^2^Department of Medicine, Nnamdi Azikiwe University, Awka Nnewi Campus, Nigeria

## Abstract

Breastfeeding is considered as the most complete nutritional source for infants because breast milk contains the essential carbohydrates, fats, proteins, and immunological factors needed for infants to thrive and resist infection in the formative first year of life. Knowledge of exclusive breastfeeding (EBF) among women is essential when promoting optimal breastfeeding practices. This cross-sectional descriptive survey assessed knowledge and intention to practice EBF and its associated factors during pregnancy among primiparous women in selected communities in Enugu State, Nigeria. A total population study that applied inclusion criteria was used to recruit 201 primiparous mothers attending their third trimester antenatal care from selected health facilities in rural and urban communities in Enugu State. A researcher-developed questionnaire was used to collect data on participants' knowledge and intention to practice EBF. Descriptive statistics of frequency, percentage, mean, and standard deviation were used to summarize categorical and continuous variables while Chi-square and Wald statistic tests predicted demographic data associated with knowledge status and intention to practice EBF of the participants. More than half (58.7%) of primiparous mothers had inadequate knowledge of EBF and only 62.7% had intention to exclusively breastfeed for 4–6 months. The Chi-square test result showed significant difference in the participants' place of residence (p = 0.024), EBF knowledge sources (p = 0.001), and EBF knowledge. The Wald statistic in Logistic regression model indicated the coefficient of age (p = 0.026), educational attainment (p = 0.046), EBF knowledge (p = 0.016), and sources of information about EBF (p = 0.027) to be significant predictors of good intention to practise EBF. Poor EBF knowledge and intention to practice in this population may be improved by combining facility-based and in-house methods of breastfeeding counseling, education, and support especially to intending and expectant mothers. Further studies are needed to be done using the multiparous women as well as assessing the effects of in-house EBF supportive-educative intervention to improve breastfeeding outcomes.

## 1. Introduction

Breast milk is considered as the most complete nutritional source for infants because it contains the essential fats, carbohydrates, proteins, and immunological factors needed for infants to thrive and resist infection in the formative first year of life [1–3]. Based on this, the World Health Organization (WHO) recommends exclusive breastfeeding for the first six months of life and continuation of breastfeeding and adequate complementary foods for up to two years of age or beyond [4]. Exclusive breastfeeding (EBF) is defined as exclusive intake of breast milk by an infant from its mother or wet nurse or expressed milk with addition of no other liquid or solid with the exception of drops or syrups consisting of vitamins, minerals supplements, or medicine and nothing else for the first six months [4]. Despite the awareness being created by various governments and non-governmental organizations on benefits of EBF, its practice remains lower than the globally recommended standard especially in developing countries [5]. According to WHO's [6] report, only 39% of all infants under six months in developing countries were exclusively breastfed for the first six months of life; 6% of infants were never breastfed; 86% and 68% of infants and children continued breastfeeding at 6-11 months and 12-23 months, respectively. In Nigeria, the Federal Ministry of Health (FMOH) [7] in her document “Saving Newborn lives Maternal and Child Health” reported that Nigeria has one of the lowest EBF rates in the African continent. The recent data indicated that the percentage of infants exclusively breastfed to the age of six months is fluctuating, from 17% in 2003 to 13.1% in 2008 and returned to 17% in 2013, while the proportion of children less than six months who received complementary foods increased from 18% to 35% in 2008 and dropped to 23% in 2013 (Nigerian Demographic & Health Survey [NDHS]) [8]. In addition, the 2013 NDHS results showed that only a third of children were breastfed within one hour of birth. Seventy-four percent of children were breastfed within one day of birth. The prevalence of early initiation of breastfeeding (within one hour) varies according to specific background characteristics, including area of residence (40% in urban areas and 29% in rural areas) [8]. Reports from some centres in Nigeria have given EBF rates at six months as follows: 26.9% in Bayelsa [9]; 58.3% in Port-Harcourt [10]; 19% in Ile-Ife [11]; 21.2% in Enugu [12].

Breastfeeding practices, including initiation and duration, are influenced by multiple interwoven factors [13]. Among these factors, decisions regarding initiation and duration of breastfeeding in low-income countries are influenced by maternal age, education, employment, parity, place of delivery, family pressure, and cultural values. [13–17] In Nigeria, these factors vary widely in various settings and even among women of the same tribe [18]. For instance, some studies have reported that parity was significantly associated with EBF [19], while some others have shown that the practice of EBF increased with increasing age of women [19–21]. Chertok et al. [22] also asserted that mother's prenatal intention to breastfeed is a consistently strong predictor of decisions to initiate and sustain exclusive breastfeeding. Besides normative expectations, personal experiences and networks of support have influence on the forms and quality of breastfeeding practices [23]. These factors exert pressure on breastfeeding mothers, thereby making their experience pleasurable or painful within time and space.

In order to achieve the benefits of optimal breastfeeding, more emphasis should be laid on transmitting knowledge to women on the practice of optimal breastfeeding. This is important because the behavior adopted by an individual on EBF is moulded partly by that individual's knowledge of EBF. Despite the available body of knowledge on breastfeeding practices in Nigeria, studies on the level of knowledge and intention to practice EBF and its predictors among primiparous women during pregnancy and how this may influence future optimal breastfeeding practices are limited in Enugu State, Nigeria. Thus, the authors attempted to assess primiparous women's EBF knowledge and intention to practice EBF as well as their associated predictors in selected rural and urban communities in Enugu State, Nigeria.

## 2. Methods

The study adopted a cross-sectional descriptive survey design. A total population study that applied inclusion criteria was used to recruit 201 primiparous mothers attending their third trimester antenatal care from selected health facilities popular in obstetric health services in selected rural and urban communities in Enugu State; namely, Comprehensive Health Centre Ahani Achi and Model Comprehensive Health Centre Inyi represent rural community while Mother of Christ Specialist Hospital and Maternity Ogui and Poly Sub-District Hospital Asata represent urban area.

### 2.1. Instrument

A researcher-developed questionnaire was used as instrument for data collection. The questionnaire comprised 19-item Antenatal Survey Form (ANSF), used to measure participants' knowledge, and intention to practice EBF. Six items elicited information on demographic characteristics of participants and four close-ended items were on knowledge, while three were on their breastfeeding intention. The instrument was validated by three experts from Department of Nursing Sciences, University of Nigeria, Enugu Campus. The instrument was pilot-tested by administering it to twenty (20) mothers from health facilities with similar characteristics. Data collected were subjected to Cronbach's alpha reliability test which gave a coefficient alpha of 0.87.

### 2.2. Ethics Approval

Ethical clearance for the study was obtained from the Ethics and Research Committee of Enugu State Ministry of Health, Enugu Nigeria (Ref. number MH/MSD/EC/0217). Administrative permit was also obtained from the administrators and medical officers' in charge of the selected health facilities used for the study, while signed informed consent was obtained from the participants.

### 2.3. Instrument Administration

Four (4) research assistants were recruited and used for data collection exercise after 30-45 minutes discussion of objectives of the study, contents of the instrument, selection of participants, and how to administer the questionnaire and interpretation of the questions in the instrument (where needed). Objectivity and confidentiality on information gathered were emphasized. Data collection was between 9.00am and 12.00pm on Tuesdays and Thursdays which were their antenatal days. The data collection exercise lasted for six months.

### 2.4. Categorization of Knowledge of Optimal Breastfeeding Practices

The assessment of correct knowledge on EBF was based on two variables:** (**a) understanding that the child is to be given breast milk only (except medications) from birth (at least one hour after birth);** (**b) identify at least five (5) benefits of EBF; and** (**c) EBF should be practiced from birth up to 4-6 months of the infant's life.

### 2.5. Method of Data Analysis

Data collected on demographic characteristics were analyzed descriptively using frequencies, percentages, mean, and standard deviation. Chi-square test of association was used to determine the association between some demographic characteristics of participants and their EBF knowledge while, in some instances, Fishers exact test was reported due to Chi-square assumption violation. A logistic regression was performed on the data to predict the logit of having good intention to practice EBF for both rural and urban women. Probability value < 0.05 was considered statistically significant. All statistical analyses were carried out with the aid of International Business Machine Statistical Package for Social Sciences [IBM SPSS] version 20.

## 3. Results

Table 1 showed the sociodemographic characteristics of the participants who fit the study inclusion criteria. Their age ranged from 16 to 42 years, with mean and standard deviation of 26.26±4.37. The participants were all Christians 201 (100.0%); predominantly of Ibo ethnic group 199(99.0%) and married 197 (98.0%); 53 (26.4%) of the participants resided in the rural area while 148 (73.6%) resided in the urban area. Most of the participants had completed secondary education 190 (94.5%), while most 166 (82.6%) were gainfully employed outside their homes.

Most of the participants 173 (86.1%) were aware of EBF; 134 (66.7%) had witnessed other mothers breastfed exclusively; 128 (63.7%) could correctly state the meaning of exclusive breastfeeding, when it is commenced, and its duration. Most known benefit of EBF identified by the participants was that of protection against infection and childhood malnutrition 134 (66.7%). Other benefits included the following: breast milk contains right amount of nutrients and water 98 (48.8%) and protection of baby against diarrhoea 88 (43.8%). Generally, majority of the participants 134 (66.7%) had poor knowledge of EBF while only 67 (33.3%) had good knowledge of EBF (Table 2).

The result in Table 3 showed that the source of information about EBF was chiefly through midwives during antenatal visits 106 (52.7%). Other sources were as follows: friends 72 (35.8%), media 58 (28.9%), and doctors 51 (25.4%). Overall, only few participants, 32 (15.9%), had much knowledge sources about EBF.

The Chi-square test result showed significant difference in the participants' place of residence (p = 0.024), EBF knowledge sources (p = 0.001), and EBF knowledge while marital status (p = 0.303), educational attainment (p = 0.161), and working status (p = 0.066) showed no significant difference (Table 4).

Table 5 showed that although 160 (79.6%) of the women had intention to start practising EBF immediately after birth but their overall intention to practice EBF was poor 115 (57.2%).

The Wald statistic in Logistic regression model indicated the coefficient of age (p = 0.026), educational attainment (p = 0.046), EBF knowledge (p = 0.016), and sources of information about EBF (p = 0.027) to be significant predictors of good intention to practise EBF. Holding other predictors constant, in predicting a woman who would have good intention, specifically for age, the odds increase by 0.91 times [95% C.I, 0.84-0.99] for 1-year increase in age. For educational attainment, the odds to have good intention increase by 6.44 times [95% C.I, 1.04-39.90], while a unit increase in knowledge and an additional knowledge source increases the odds of having good intention by 1.21 times [95% C.I, 1.04-1.41] and 1.31 times [95% C.I, 1.03-1.67], respectively. For the coefficient of working status (p = 0.411) and residence (p = 0.727), the Wald statistic revealed no significance (Table 6).

## 4. Discussion

The participants in this study had similar antenatal demographic characteristics with those that were reported from other health facilities in Nigeria in terms of age, educational attainment, employment status, and marital status. The age range and mean age of the study group (16-42 years and 25.49-27.34 years, respectively) did not differ much from the age range and mean (19-51 years and 35 years, respectively) reported by Adeyinka et al. [5]. Most of the participants had formal education, majorly secondary education for the rural groups, and tertiary education for the urban groups. Women with some formal education are more likely to seek medical care, be better informed about their children's nutritional requirements, and adopt improved healthy practices. This showed that women are moving with trends in educational system in Nigeria. For employment status, most of participants (83.6%) were working, either employed in a paid job or self-employed. Predominantly 98.0% of the women were in a committed relationship (married) and 86.3% planned to return to work after giving birth, mainly after three to four months (62.1%).

In this study, more than half (58.7%) of primiparous mothers had inadequate knowledge of EBF and only 62.7% had intention to exclusively breastfeed for 4–6 months. This is low given that most primiparous women had formal education and had attended antenatal clinic more than once. Ordinarily, one would think that the participants' visits and exposure during antenatal clinics would improve their knowledge of EBF. This assumption was, however, not reflected in the findings. It is likely that some of these women might not have accessed antenatal care services where appropriate key messages on EBF are usually delivered or they might be among the group that visit the antenatal clinics after the routine breastfeeding education must have been delivered by the health personnel. These findings are in keeping with other studies [20–23]. More so, the poor knowledge exhibited by the participants might be associated with their parity since this is the first time they are exposed to experiences of motherhood and quite a good number of them claimed they had not witnessed other mothers breastfeeding exclusively. Results of the study further showed that participants' place of residence (rural versus urban) had significant association with their EBF knowledge. This was obvious as many as 79.2% from rural area had poor knowledge of EBF. Similarly, other sources of information might have influenced urban women's EBF knowledge since their knowledge sources varied widely compared to the rural women. This was also found in a study by Asfaw et al. [24] in Central Ethiopia where 30.4% of women had also received information from the media and 18.4% from their friends. This shows that other sources of communication can be a good source of knowledge on appropriate breastfeeding practices. Thus, the country needs to come up with a communication strategy that will be simple and user friendly, especially for the women in rural communities on EBF in Nigeria. This can then be used both at facilities and in home environment to contribute in improving poor breastfeeding practices and nutrition indicators among infants and children as a whole.

Breastfeeding practices are influenced by multiple interwoven factors [13–16]. Results of the study indicated the coefficient of age (p = 0.026), educational attainment (p = 0.046), EBF knowledge (p = 0.016), and sources of information about EBF (p = 0.027) to be significant predictors of good intention to practise EBF. However, the age group of most mothers in this study was between 21 and 30 years who seem to be more energetic, serious, and more educated and as such more eager to get information that will enhance child survival and development. Experience has also shown that underaged mothers seldom have less time to utilize exclusive breastfeeding unlike matured mothers who appreciate the cordiality that exists between mother and infants as a result of exclusive breastfeeding practices.

The result further indicated that educational background of mothers was found to be a significant predictor of good intention to practise EBF. To a large extent, educated women tend to follow the antenatal instructions to the letter, thereby changing their attitudes towards practice of EBF. This finding agrees with many other studies [23, 25] that reported on the influence of mothers' educational level on their decision to exclusively breastfeed. Mothers who had no formal education were more unlikely to practice exclusive breastfeeding than their peers with higher education as mothers with no education tend not to be well informed about the benefits of exclusive breastfeeding as compared to their counterparts with higher education [23]. In addition, EBF knowledge and sources of information about EBF were found to be significant predictors of good intention to practise EBF. This finding is in line with studies by Agho et al. [21] and Qureshi et al. [23] that reported that mothers who accessed antenatal care services during pregnancy were more likely to practice exclusive breastfeeding as appropriate key messages were usually delivered during antenatal care services. Researchers noted that if pregnant women or mothers are educated and counseled properly on the benefits of exclusive breastfeeding, they are more likely to practice EBF than their peers who are not counseled. Similarly, varied sources of information on EBF such as midwives, doctors, friends, media, and peers amongst others had also strengthened their EBF knowledge, thereby influencing their decisions to practice EBF.

The implication of these findings is that, despite the awareness being created by WHO and other organizations on the benefits of EBF, statistics are still indicative of inadequate knowledge and low patronage among women. The authors strongly believe of the need for continuous education and reiteration of the benefits of EBF by midwives and other health workers at every point of contact with mothers so as to improve on their exclusive breastfeeding practice. Establishment of breastfeeding support groups should be encouraged.

## 5. Conclusion

Findings of this study revealed that primiparous women's knowledge on EBF and intention to practice it is still low. There was significant difference in the participants' place of residence (rural versus urban), EBF knowledge sources, and EBF knowledge, while the maternal age, educational attainment, EBF knowledge, and sources of information about EBF were found to be significant predictors of good intention to practise EBF.

## Figures and Tables

**Table 1:** Sociodemographic characteristics of the participants (n = 201).

	**Frequency**	**Percentage**	**Range**	**M±SD**
**Age (in years)**			16-42	26.26±4.37
< 20	18	9.0		
21-25	68	33.8		
26-30	87	43.3		
> 30	28	13.9		
**Religious affiliation**				
Christian	201	100.0		
**Tribe**				
Igbo	199	99.0		
Ibibio	2	1.0		
**Residence**				
Rural	53	26.4		
Urban	148	73.6		
**Marital status**				
Married	197	98.0		
Single	4	2.0		
**Highest educational attainment**				
No formal	3	1.5		
Primary	8	4.0		
Secondary	77	38.3		
Tertiary	113	56.2		
**Working status**				
Working	166	82.6		
Not working	35	17.4		

**Table 2:** Knowledge of exclusive breastfeeding (n = 201).

**Variables **	**Frequency**	**Percentage**
*∗*Awareness of EBF		
Yes	173	86.1
No	28	13.9
**Have witnessed other mothers breastfed Exclusively**		
Yes	134	66.7
No	67	33.3
**∗** **Definition of EBF**		
Correct definition of EBF	128	63.7
Incorrect definition of EBF	73	36.3
**Time of initiation of EBF**		
Within 1 hour after birth	128	63.7
After 24 hours of birth	63	31.3
I don't know	10	5.0
**∗** **Perceived benefits of EBF**		
Protection against infection and childhood malnutrition	134	66.7
Contains right amount of nutrients and water	98	48.8
Protection of baby against diarrhoea	88	43.8
Strengthens mother-child bonding	72	35.8
Economical and readily available	59	29.4
Aids in child spacing	33	16.4
Protects the mother against cancers	26	12.9
Others *(intelligent & healthy baby) *	32	16.4
**Overall knowledge**		
Good *(knowledge score > 50*%)	67	33.3
Poor *(knowledge score ≤ 50*%)	134	66.7

*∗*Variables used to assess EBF knowledge.

**Table 3:** Sources of information about exclusive breastfeeding (n = 201).

**Sources of information about EBF**	**Frequency**	**Percentage**
Midwife during antenatal visit	106	52.7
Friends	72	35.8
Media	58	28.9
Doctor	51	25.4
Mother	42	20.9
Husband	17	8.5
Others (marriage counselor, profession, relative)	19	9.5
**Overall EBF knowledge sources**		
Much (knowledge source > 50%)	32	15.9
Few (knowledge source ≤ 50%)	169	84.1

**Table 4:** Chi-square test of association between demographic variables and EBF knowledge (n = 201).

**Variables**	**Knowledge of EBF**			**Chi-Square**	**df**	**p-value**
	**Good** **(n = 67)**	**Poor** **(n = 134)**	**Total** **(n = 201)**			
**Age**				5.541	3	.136
≤ 20	3(16.7)	15(83.3)	18			
21-25	20(29.4)	48(70.6)	68			
26-30	36(41.4)	51(58.6)	87			
> 30	8(28.6)	20(71.4)	28			
**Marital status**				-	-	.303*∗*
Married	67(34.0)	130(66.0)	197			
Single	0(0.0)	4(100.0)	4			
**Place of residence **				5.125	1	.024^a^
Rural	11(20.8)	42(79.2)	53			
Urban	56(37.8)	92(62.2)	148			
**Educational attainment**				3.656	2	.161
≤ Primary	3(27.3)	8(72.7)	11			
Secondary	20(26.0)	57(74.0)	77			
Tertiary	44(38.9)	69(61.1)	113			
**Working status**				3.390	1	.066
Working	60(36.1)	106(63.9)	166			
Not working	7(20.0)	28(80.0)	35			
**EBF knowledge sources**				25.441	1	.001^a^
Much	23(71.9)	9(28.1)	32			
Few	44(26.0)	125(74.0)	169			

*∗* Fishers Exact Test p-value reported (due to Chi-Square assumption violation).

^a^Significant.

**Table 5:** Intention to practice exclusive breastfeeding (n = 201).

	**Frequency**	**Percentage**
**Intention in starting EBF**		
Immediately after birth	160	79.6
Later	2	1.0
Undecided	39	19.4
**Intention in length of EBF**		
< 6 months	80	39.8
≥ 6 months	89	44.3
Undecided	32	15.9
**Overall intention**		
Good *(if intention is to start EBF immediately & to last *≥* 6 months)*	86	42.8
Poor *(if otherwise)*	115	57.2

**Table 6:** Logistic regression model coefficients on intention of exclusive breastfeeding.

							95% C.I. for EXP(B)	
	B	S.E.	Wald	df	p-value	Exp(B)	Lower	Upper
**Age**	-.094	.042	4.983	1	.026	.911	.839	.989
**Educationalattainment**			6.176	2	.046			
Secondary	1.154	.919	1.577	1	.209	3.171	.523	19.215
Tertiary	1.863	.930	4.012	1	.045	6.444	1.041	39.902
**Workingstatus** (Not working)	.360	.439	.675	1	.411	1.434	.607	3.388
**Residence** (Urban)	.139	.398	.122	1	.727	1.149	.527	2.507
**EBF knowledge score**	.191	.079	5.857	1	.016	1.210	1.037	1.413
**EBF knowledge sources**	.273	.123	4.896	1	.027	1.314	1.032	1.673
Constant	-.647	1.370	.223	1	.637	.524		

Predictors: age, educational attainment, working status, residence, EBF knowledge & EBF sources of information.

Variable type used: numeric (age, EBF knowledge score, & sources of information); categorical (education, working status, & residence).

Reference category: education (≤ primary); working status (working); residence (rural).

## Data Availability

The raw data used to support the findings of this study are available from the corresponding author upon request.

## Additional Points

*Recommendations. *(i) Emphasis should be laid on making sure that every pregnant woman attending antenatal care receives counseling and support on optimal breastfeeding practices. (ii) Other channels of disseminating information on optimal breastfeeding practices for child growth and survival, such as the social media, should also be used. (iii) Combining facility-based and in-house methods of counseling might help in increasing the number of women who understand the importance of EBF and adhere to optimal breastfeeding. (iv) Intervention programmes on the awareness and benefits of EBF among mothers should be disseminated at the community level and should involve important key players such as community leaders, husbands, mothers-in-law, and grandmothers as they often influence the decision of a mother to exclusively breastfeed. (v) The study was only on primiparous women; thus the findings cannot be generalized for multiparous women. Further studies are needed to be done using the multiparous as well as assessing the effects of in-house EBF supportive-educative intervention to improve breastfeeding outcomes.
